# Bio-Based Carbon Materials for High-Performance Supercapacitors

**DOI:** 10.3390/nano12172931

**Published:** 2022-08-25

**Authors:** Penghui Li, Chi Yang, Caiwen Wu, Yumeng Wei, Bo Jiang, Yongcan Jin, Wenjuan Wu

**Affiliations:** 1 Jiangsu Co-Innovation Center of Efficient Processing and Utilization of Forest Resources, Nanjing Forestry University, Nanjing 210037, China; 2 College of Light Industry and Food Engineering, Nanjing Forestry University, Nanjing 210037, China

**Keywords:** lignosulfonate, polyaniline, doping, electrode material, electrochemical performance

## Abstract

Lignin, one of the components of natural plant biomass, is a rich source of carbon and has excellent potential as a valuable, sustainable source of carbon material. Low-cost lignosulfonate (LS) doped with polyaniline (PANI) has been used as a precursor to produce porous carbon. LS has a highly dispersed and sparse microstructure and can be accidentally doped with S atoms. N and S double-doped carbon can be directly synthesized with abundant mesopores and high surface area in a lamellar network using PANI as another doping source. This study explored the optimal conditions of LS/PANI material with different amounts of lignosulfonate and different carbonization temperatures. When the amount of lignosulfonate was 4 g and the carbonization temperature was 700 °C, graded porous carbon was obtained, and the electrochemical performance was the best. At 0.5 A/g, the specific capacitance reached 333.50 F/g (three-electrode system) and 242.20 F/g (two-electrode system). After 5000 charge/discharge cycles at 5 A/g, the material maintained good cycling stability and achieved a capacitance retention rate of 95.14% (three-electrode system) and 97.04% (two-electrode system). The energy and power densities of the SNC700 samples were 8.33 Wh/kg and 62.5 W/kg at 0.25 A/g, respectively, values that meet the requirements of today’s commercially available supercapacitor electrode materials, further demonstrating their good practicality. This paper provides an efficient double-doping method to prepare layered structures. Porous carbon is used for electrochemical energy storage devices.

## 1. Introduction

As technology advances and people’s living standards improve, energy is being consumed, fossil fuels are in short supply, and environmental problems are becoming more and more prominent. Based on this, it is essential to research and create new high-performance green energy storage and conversion devices [1]. It is well known that supercapacitors (SCs) and electrochemical double-layer capacitors (EDLCs) both have fantastic energy storage performance, and they can provide sufficient energy for high-power (10 kW/kg) applications with fast charging/discharging [2]. Supercapacitors have excellent applications in electronic payments as well as in transportation. However, most of them have low performance (e.g., low energy density) and generally high prices, preventing their applications from being scaled up [3]. Currently, the most critical electrode material for promoted supercapacitors is porous carbon, which has a high specific surface area (SSA) and a vast pore size distribution, thus allowing the electrostatic charge accumulation/release at the electrode/electrolyte interface to store energy [4]. Porous carbon has a low cost and easy preparation process, excellent chemical stability, and high conductivity/ionic conductivity, making it suitable for SC electrode materials [5]. Therefore, it is urgent to develop porous carbon materials with low production costs to reduce the energy storage costs of supercapacitors [6].

Among many electrode materials, conductive polyaniline (PANI) is worthy of our attention because of its high theoretical specific capacity, high stability, low cost, high conductivity (close to 2.0 S/cm), easy polymerization in aqueous media, and easy doping/dedoping, making it one of the ideal polymers [7,8]. However, the unsatisfactory mechanical properties of PANI hinder its development. Compared with polyaniline, the advantages of functionalized carbon materials are manifested in excellent physical and chemical properties (such as accessibility to active sites, ideal specific surface area, porous structure, and good electrical conductivity). However, the main disadvantage of porous biomass carbons is their relatively low bulk density (<0.5 g/cm^3^) because of their extremely high surface area (~1000–3000 m^2^/g), which limits the bulk capacitance (low to 60 F/cm^3^) [9]. In order to be able to create better electrode materials and achieve positive synergistic effects, the method of compounding carbon materials with polyaniline came into being.

The production of carbon materials from green, sustainable, and renewable sources has attracted a great deal of attention [10]. Among renewable biopolymers, lignin, the second largest natural polymer after cellulose, is a three-dimensional aromatic biopolymer containing phenylpropane units linked together by various bonds to form a highly complex structure. Pulping in the paper industry generally uses sulfuric acid (kraft process) or sulfurous acid (sulfite process) to remove lignin, which converts insoluble lignin into soluble lignosulfonate. Therefore, lignosulfonate is a significant and substantial by-product of the modern paper industry, often commonly burned to recover sulfur and generate heat [11]. Lignosulfonate is pyrolytically decomposed into sulfates, which can act as porogenic agents. In addition, lignosulfonate contains phenolic hydroxyl and carboxyl groups that can react with alkali metal salts, and the metal sites partially bound to them can be used to generate inorganic templates (sulfates, oxides, etc.), which will be of great benefit to the pore formation process during the carbonization of lignosulfonate [6]. Combining the above viewpoints, lignosulfonate is very suitable as a precursor of carbonaceous materials [12,13]. Wang et al. [14] used chemical oxidative polymerization to prepare PANI/lignin composites with interpenetrating networks; the conductivity of the best PANI/lignin composites was 3.2 S/cm, and PANI was only 1.4 S/cm. At a high current density of 30.0 A/g, the specific capacitance of the composite reached 284.4 F/g, while that of PANI was 123.1 F/g. This particular interpenetrating network facilitates ion diffusion within the PANI material, and the three-dimensional network of lignin can efficiently reduce the mechano-mechanical damage caused by the volume change of PANI during charge/discharge cycles.

Regarding the choice of activation mode, the advantage of chemical activation is greater than that of physical activation. Common activators include KOH, K_2_CO_3_, KCl, etc., mixed with carbon materials and carbonized to prepare porous carbons in an inert atmosphere [15]. Among them, carbon materials activated with KOH have large SSA (>2000 m^2^/g) [16]. Doping various heteroatoms (nitrogen, oxygen, sulfur, etc.) into the carbon framework can provide additional pseudocapacitance and increase the electronic conductivity and surface wettability of the carbon structure, which can greatly improve the carbon capacitance feature. The most common doping element is the nitrogen atom. After doping, the nitrogen atom replaces the original carbon atom position, and the graphite structure also changes, which improves the reactivity [17]. The introduction of nitrogen into porous carbon increases the number of pores, increases nitrogen-containing functional groups, increases the specific surface area, and enhances the material capacitance [18]. Park et al. [19] used lignosulfonate as the carbon precursor, and after treatment with urea, N functional groups were successfully introduced into the porous carbon surface with increased surface area. There are also more doping elements, such as sulfur. The sulfur atom has electron-withdrawing properties, which can change the electronic structure and electron density balance of carbon atoms and generate electroactive centers on the material’s surface, improving the electrocatalytic and capacitive performance of carbon materials [20]. Demir et al. [21] converted lignosulfonate into doped carbon with a sulfur content of 3.2 wt% by in situ hydrothermal carbonization and thermal annealing. The resulting material showed a high surface area of up to 660.0 m^2^/g. After doping sulfur, the electrode material offers high stability and high electrochemical activity, suitable for applying to supercapacitors. At the current density of 0.5 A/g, the capacitance reaches 225.0 F/g.

Co-doping of two heteroatoms, such as nitrogen and sulfur, can further improve the performance of porous carbon through a synergistic effect and can be applied to supercapacitors to obtain good performance. Tian et al. [22] used lignosulfonate as carbon and sulfur precursors and polyaniline as a nitrogen precursor. They prepared nitrogen–sulfur co-doped three-dimensional hierarchical porous carbons with KOH activation, resulting in a hierarchical porous carbon structure. It has high specific capacitance and strong cycling stability when used as a supercapacitor electrode. Lignosulfonate is used as a precursor because the sulfonate functional group has a high sulfur content. During the activation phase, the C–S–C structure can increase the specific surface area and generate ultra-micropore, micropore, and mesopore structures [23]. Inspired by this idea, the authors speculate that chemical activation by pyrolysis using N-rich chemicals (urea, etc.) will produce nitrogen-containing carbon materials. Lignosulfonates are also relatively high in sulfur, and biomass resources are abundant. The ready availability, as well as the interpenetrating porous network formed with polyaniline, leads to high-performance supercapacitors. Based on the above points, we propose to use lignosulfonate as a sustainable carbon precursor with polyaniline to prepare hierarchical porous carbons, which will achieve excellent results as carbon materials for supercapacitor electrodes.

## 2. Experimental Section

### 2.1. Materials and Reagents

Lignosulfonate (LS, Mw = 10,000) and N-Methylpyrrolidone were purchased from Aladdin Reagent Company (Shanghai, China). Conductive carbon black, polyvinylidene difluoride (PVDF), aniline (used after two vacuum distillations), and ammonium persulfate were purchased from Macklin Reagent Co., Ltd. (Nanjing, China); concentrated hydrochloric acid (Analytical Grade), nickel foam, and other chemicals were purchased from Sinopharm Chemical Reagent Co., Ltd. (Shanghai, China). Laboratory water was ultrapure water.

### 2.2. Preparation of LS/PANI

**Figure 1:**
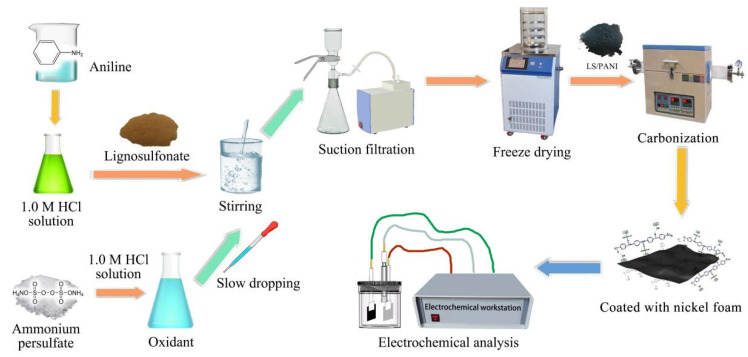
Flow chart of LS/PANI electrode material preparation.

The preparation process of the lignosulfonate/polyaniline (LS/PANI) precursor is shown in Figure 1. First, 0.91 mL of purified aniline was uniformly dispersed in 100 mL of 1M HCl, and 1 g, 2 g, or 4 g of lignosulfonate was added to the aniline solution and stirred magnetically at 0–4 °C for 2 h. Then, 2.28 g of ammonium persulfate was weighed into 100 mL of 1 M HCl and stirred for 24 h to obtain a uniform solution. The solution was put into the reaction system at a molar ratio of 1:1 between aniline and ammonium persulfate and stirred magnetically at 4 °C for 24 h. The reaction product was filtered, washed repeatedly with deionized water and ethanol, freeze-dried for 48 h, and vacuum-dried to a constant weight to obtain a dark green solid. As a reference, pure PANI material without added lignosulfonate was prepared by the same method.

### 2.3. Preparation of Porous O/N/S Co-Doped Activated Carbon Materials

**Figure 2:**
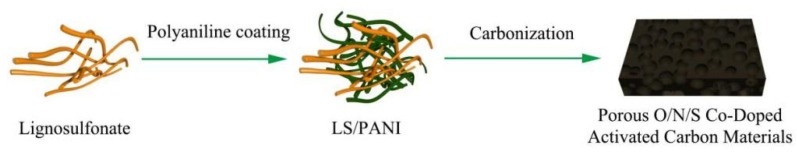
Preparation process of porous O/N/S co-doped activated carbon materials.

Nitrogen/sulfur co-doped activated carbon materials were prepared by a two-step calcination method (see Figure 2). The first step was pre-carbonization. The above lignosulfonate/polyaniline composites were dried to constant weight; then, 2 g was taken and carbonized in a tube furnace at 400 °C for 1.5 h, and the obtained powder samples were ground thoroughly. The second step was activation. The ground powder was mixed with the solid activator KOH according to an impregnation mass ratio of 1:1, stirred for 5 h until homogeneous, and dried in a vacuum drying oven at 80 °C for 12 h. The dried samples were heated to 500 °C, 700 °C, and 900 °C in a tube furnace at a heating rate of 5 °C/min under a N_2_ atmosphere, held for 2 h, and then cooled to room temperature. The treated material was washed in 1 M HCl to remove the KOH activator and then repeatedly washed with distilled water to a neutral pH. The washed residue was dried to a constant weight in a blast oven at 105 °C, which was the O/N/S co-doped activated carbon material. After subsequent experiments, it was found that 4 g of lignosulfonate was the optimal addition amount. In this experiment, the charring temperature was investigated with 4 g of lignosulfonate addition, and the charring temperatures were 500 °C, 700 °C, and 900 °C, labeled SNC500, SNC700, and SNC900, respectively.

### 2.4. Material Characterizations

The surface morphology of the samples was characterized using field-emission scanning electron microscopy (SEM, FEI Quanta 200, Hillsboro, OR, USA) and transmission electron microscopy (TEM, JEM-1400, JEOL, Tokyo, Japan). A combined multifunctional horizontal X-ray diffractometer (XRD, Ultima IV, Rigaku, Tokyo, Japan) (copper target λ = 0.15406 nm, voltage 40 kV, current 200 mA, 2θ = 5–60°, and scan rate 15°/min) was used for polymer crystal structure analysis. X-ray photoelectron spectroscopy (XPS, AXIS Ultra DLD, Kratos, Manchester, UK) was used to study the surface chemical properties of the materials, including the relative contents of elements and the types and relative contents of chemical functional groups. FT-IR and Raman characterizations were carried out with an infrared spectrometer (FT-IR Bruker VERTEX 80V, Bremen, Germany) and laser Raman spectrometer (Raman Thermo DXR532, Thermo Fisher Scientific, Waltham, MA, USA), respectively. Surface area and pore size distribution were estimated from isotherms of nitrogen adsorption–desorption measurements by Brunauer–Emmett–Teller analysis (BET, QUADRASORB-EVO, Quantachrome Instruments, Boynton Beach, FL, USA). The electronic conductivity of carbon materials was measured by the conventional four-probe technique. Under a pressure of 20 MPa, the sample was pressed into a disk of 2 mm thickness.

### 2.5. Preparation of Working Electrodes

The NC and SNC samples, conductive carbon black, and binder (polyvinylidene fluoride, PVDF) were mixed in a weight ratio of 8:1:1. Subsequently, 1–2 drops of *N*-methylpyrrolidone were added and uniformly applied to the pre-treated nickel foam with a loading of approximately 3–5 mg/cm^2^ active material per electrode. The mixture was then pressed onto the nickel foam at a pressure of 10 MPa and dried under vacuum at 80 °C for 12 h to complete the working electrode preparation.

The electrode plates were assembled into a symmetrical supercapacitor, the electrode shell was CR2032 type, and the positive and negative electrode materials were the same material, both of which were carbon materials prepared in this experiment. Two pre-treated nickel foams with similar active materials were selected at a pressure of 10 MPa. Tablets were pressed using 6 mol/L KOH aqueous solution as an electrolyte and encapsulated in a 2032 electrode shell with a packaging machine, and electrochemical tests were carried out after standing for 12 h.

### 2.6. Electrochemical Measurements

The electrochemical performance was tested using a CH-instruments CHI660E electrochemical workstation in a three-electrode system and a 6 mol/L KOH electrolyte solution. The platinum sheet electrode was used as the counter electrode, and a Ag/AgCl electrode was the reference electrode. The cyclic voltammetry curves (CV) of the working electrodes were tested in a voltage range of −1 V to 0 V, and different sweep rates (10, 20, 30, 50, and 100 mV/s) were used to investigate the multiplicity characteristics. Constant current charge/discharge curves (GCD) were tested in the voltage range of −1 V to 0 V, and the specific capacitance of the electrode material was calculated according to Equation (2), obtained by testing at different current densities (0.5, 1, 3, 5, and 10 A/g). Electrochemical impedance spectroscopy was carried out on carbon electrodes in the frequency range 10^−2^–10^5^ Hz with an amplitude of 5 mV and an initial open-circuit voltage. The cycle life of the carbon material electrodes was investigated by conducting 5000 constant current charge/discharge tests at a current density of 5 A/g.

The linear charge/discharge curve formula is shown in Equation (1):

(1) $$C_{m\;}=C/m=I\Delta t/\left(m\Delta v\right)$$

In the formula, C_m_ (F/g) is the specific capacitance; m (g) is the electrode material load; I (A) is the charge and discharge current; Δt (s) is the charge and discharge time; and ΔV (V) is the voltage window.

The nonlinear charge/discharge curve formula is shown in Equation (2):

(2) $$C_{m}=\frac{2\times I\times S}{m\times \Delta U^{2}}=\frac{2\times I\int_{{t(U}_{\max})}^{{t(U}_{\min})}U(t)\mathrm{dt}}{m\times \Delta U^{2}}$$

In the formula, I (A) is the charge and discharge current; S (Vs) is the integral area under the discharge curve; m (g) is the active material of the electrode; t (U_max_) is the discharge start time (s); t (U_min_) is the discharge end time (s); and ΔU (V) is the voltage range after deducting the voltage drop.

For symmetrical supercapacitors, the specific capacitance of the electrode material is four times higher than the specific capacitance of the battery. The energy density and power density are expressed by the following Equations (3) and (4):

(3) $$E=\frac{1}{4}\times I\int_{0}^{t}Udt\times \frac{1}{3.6}$$

(4) $$P=\frac{3600\times E}{\Delta t}$$

where C (F/g) is the specific capacitance of the supercapacitor, *E* (Wh/kg) is the energy density of the supercapacitor, ∆*U* (V) is the capacitor (or cell) voltage, *P* (W/kg) is the power density of the supercapacitor, and *t* (h) is the discharge time.

## 3. Results and Discussion

### 3.1. Morphological Analysis

#### 3.1.1. Scanning Electron Microscopy (SEM)

**Figure 3:**
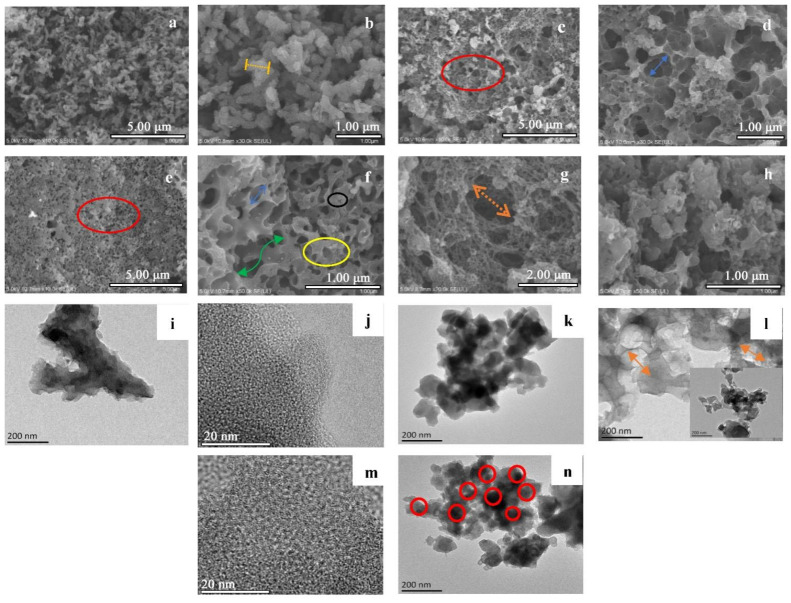
SEM images: (**a**,**b**) NC700, (**c**,**d**) SNC500, (**e**,**f**) SNC700, and (**g**,**h**) SNC900; TEM images: (**i**,**j**) NC700, (**k**) SNC500, (**l**,**m**) SNC700, and (**n**) SNC900.

In addition to examining the vital role of LS in the synthesis of layered porous carbon, the morphology of the carbon material changed considerably at different carbonization temperatures. As shown in Figure 3a,b, pure polyaniline formed fibrous morphologies that were twisted, interpenetrated, or twisted and cross-linked with each other after carbonization at 700 °C [24]. After KOH expansion activation, it was found that the activator did not penetrate the pure polyaniline material, and the morphology of polyaniline did not change after carbonization. In contrast, the surface morphology of the material changed significantly after lignosulfonate incorporation at the three charring temperatures (500 °C, 700 °C, and 900 °C), as can be seen in Figure 3c–h: with increasing calcination temperature, the morphology of the products after charring varied for SNC500, SNC700, and SNC900. The 500 °C material had large pores (>50 nm) (red box in Figure 3c,e) and formed more frequently, while for material carbonized at 700 °C, as shown in Figure 3e, the increased dehydrogenation and pore expansion by the activator caused polymer cleavage to generate more pores of different sizes. The large pores tended to form vertically or laterally interconnected channels (green arrows in Figure 3f). Lignosulfonate functional groups generate more sulfur material and introduce more defects in the carbon skeleton [25].

During the carbonization process, KOH will readily decompose and react with the resulting carbon and carbon dioxide:

(5) $$2\mathrm{KOH}\to K_{2}O+H_{2}O$$

(6) $$K_{2}O+{\mathrm{CO}}_{2}\to K_{2}{\mathrm{CO}}_{3}$$

(7) $$4\mathrm{KOH}+C\to K_{2}{\mathrm{CO}}_{3}+K_{2}O+2H_{2}$$

Subsequently, KOH readily generates K_2_CO_3_, which is itself an activator, so the effect is greatly enhanced by using KOH as an activator [26]:

(8) $$K_{2}O+C\to 2K+\mathrm{CO}$$

(9) $$K_{2}{\mathrm{CO}}_{3}+C\to 2K+3\mathrm{CO}$$

As shown in Figure 3f,g, high-temperature carbonization from 700 °C to 900 °C causes potassium carbonate or potassium oxide to react with the resulting carbon, after which the evaporation of metallic potassium expands the pore size [27,28]. Potassium metal can swell the internal structure between carbons, expanding on the original [29]. In addition, potassium can reduce the surface tension on the carbon surface and produce more reactive sites [30]. Polyaniline can grow reactive sites, resulting in a thinner fibrous structure and an increased specific surface area, generating high-quality porous carbon [24]. During activation, these sulfur functional groups create additional pores (yellow box and black box in Figure 3f). The high-temperature conditions at 900 °C resulted in the collapse of the macroporous structure of the material, with the surface showing as shown in Figure 3g,h. The layers are stacked in a disordered state with some degree of agglomeration, the surface and interior are corroded, and the internal structure is a tightly connected three-dimensional porous network structure that provides support for the nucleation of the porous carbon skeleton [31].

As seen in Figure 3i, pure polyaniline was carbonized as large agglomerated particles with a diameter of about 150 nm and a length of about 600 nm, which is consistent with the SEM analysis. LS was better dispersed with polyaniline. In Figure 3k,l,n, individual nanorods or nanoparticles can be found, the surface of which is partially covered by smaller spherical lignosulfonate particles, and the fibers became thinner after lignosulfonate incorporation, with a diameter of 50–100 nm [32]. This may be due to volume shrinkage caused by the thermal degradation of LS/PANI to charcoal [7]. Nanoparticles with a diameter of about 30 nm were uniformly dispersed in the laminated carbon structure, forming an interpenetrating network (Figure 3l) [33]. As seen in Figure 3l,m, the hexagonal ring surface of the SNC700 carbon atoms formed a disordered, irregular lamellar structure with crystal defects [34]. Figure 3m shows ordered and disordered graphitic carbon that can provide defects for the adsorption and embedding of ions or electrons and active sites. Furthermore, the lattice defects are more numerous than the undoped polyaniline carbon material with lignosulfonate. Figure 3j shows the structure of pyrrole nitrogen. In contrast, a further increase in pyrrole nitrogen content can be seen in Figure 3m, where the rise in pyrrole nitrogen content promotes the electrochemical properties of the nitrogen-doped carbon. Upon comparison, it was found that the synthesis of polyaniline in the presence of lignosulfonate resulted in similar nanorod agglomerates, but with smaller circular lignosulfonate particles embedded in an interpenetrating network, and good dispersion and compatibility of the polyaniline nanoparticles due to the high number of nucleation sites in layered porous carbon [14]. At the same time, the morphology of the porous carbon underwent a significant change with the increase in carbonization temperature, and the lamellar phenomenon was most serious in the SNC900 material, probably due to the thermal decomposition of polyaniline and the collapse and disordered stacking of a part of it.

#### 3.1.2. FT-IR Analysis

**Figure 4:**
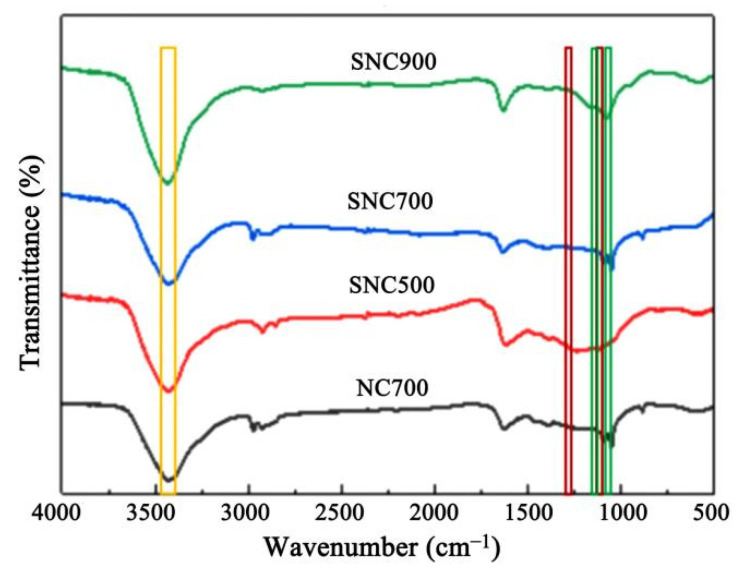
FT-IR spectra of NC and SNC at different carbonization temperatures.

In Figure 4, it can be seen that both SNC and NC have similar IR characteristics at different carbonization temperatures. It can be seen in the figure that the characteristic absorption peaks of O–H and N–H appear at 3430 cm^−1^, 1224 cm^−1^, and 1100 cm^−1^, which correspond to the characteristic absorption peaks of C–O and C=O bonds, respectively. The sulfonic acid group’s symmetric and asymmetric vibrational absorption peaks are at 1039 cm^−1^ and 1139 cm^−1^, where 1039 cm^−1^ is a mixed vibrational peak of the sulfonic acid group and alkyl ether bond.

As the pyrolysis temperature increases, the aniline-derived C=C and C–N functional groups show a significant red shift and weakening of the spectral peaks corresponding to the absorption wavelengths at 1626 cm^−1^ and 1382 cm^−1^ due to the escape of carbon and nitrogen components [35].

#### 3.1.3. XRD Analysis

**Figure 5:**
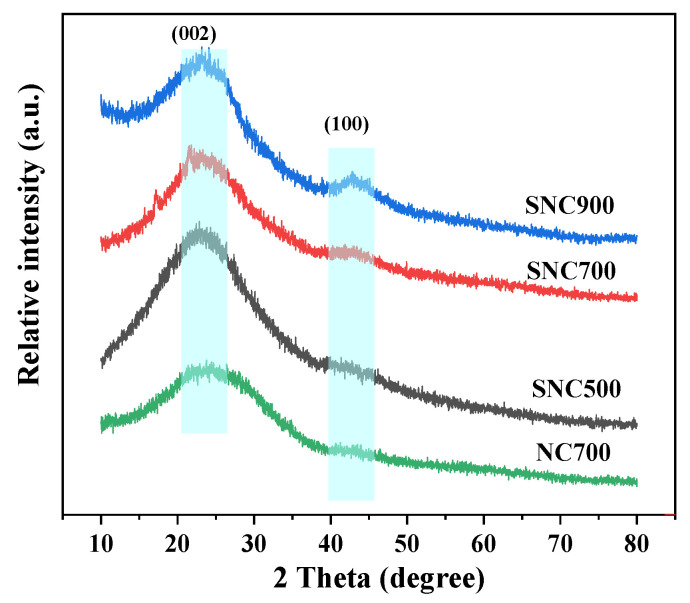
XRD patterns of NC and SNC.

The structure of the prepared carbon was investigated by XRD (Figure 5), and it can be noted that the samples calcined at different temperatures exhibit almost the same characteristic pattern. The two diffraction peaks at 23.5° and 43.3° correspond to the (002) and (100) crystallographic planes, respectively, indicating a typical amorphous carbon material.

When polyaniline is carbonized, its structure is disrupted under high-temperature conditions, and the composite is an amorphous carbon material [36]. The broad peak at 2θ = 43.3° represents the in-plane diffraction of the graphite crystalline phase. At a calcination temperature of 900 °C, the structural defects of the material increase, and the graphite flake structure is the most abundant. The peak shapes of SNC700 and NC700 are more similar, indicating that the doping of lignosulfonate does not change the overall structure of the material, and the calcination temperature significantly influences the surface morphology of the material, which in turn affects the properties of the carbon materials.

In contrast, the carbon material obtained at a calcination temperature of 500 °C has only a broad diffraction peak at 23.5°, indicating that the sample has the most amorphous shape. As can be seen in Figure 5, the polyaniline diffraction peaks are mainly between 2θ = 10–30°. The two diffraction peaks at 20.38° and 25.1° represent the characteristic peaks of polyaniline periodically parallel to the crystal plane of the leading chain group (100) and perpendicular to the crystal plane of the leading chain group (110), respectively; after activation and high-temperature carbonization, the prominent diffraction peaks destroy the crystalline structure of polyaniline.

#### 3.1.4. Raman Spectroscopy Analysis

**Figure 6:**
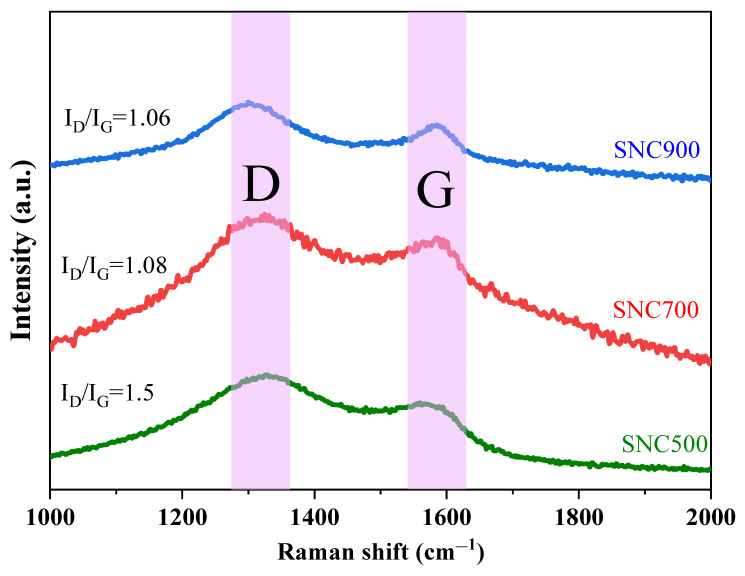
Raman spectra of SNC at different carbonization temperatures.

Two distinguishable broad peaks are present in the Raman spectrum at 1327 cm^−1^ and 1575 cm^−1^. They represent the disordered state and graphitized structure of the lignosulfonate/polyaniline carbon material, defined as the D-band and G-band, respectively [37]. It can be seen in the Figure 6 that at the higher carbonization temperature, the intensity of the D-band is higher than that of the G-band in both spectral bands, indicating more amorphous structures at all three carbonization temperatures. Among them, the highest degree of graphitization is shown by the low intensity of disordered carbon at the carbonization temperature of 900 °C. The intensity ratio (I_G_/I_D_) depends on the degree of graphite structure and can be used to measure structural defects and impurities in carbon materials. In the present study and similar studies on lignin carbon materials, I_D_/I_G_ is approximately equal to 1 [37], indicating the presence of both ordered graphitic domains and a large number of disordered regions.

The characteristic band appearing at 1566–1635 cm^−1^ represents the stretching vibration of the C=C quinone ring and the benzene ring, and the band at 1280–1375 cm^−1^ corresponds to the stretching vibration of the C–N^+^ mode of the nitrogen cation radical, confirming the doping of the N element in the composite, which is consistent with the XPS results.

#### 3.1.5. XPS Analysis

**Figure 7:**
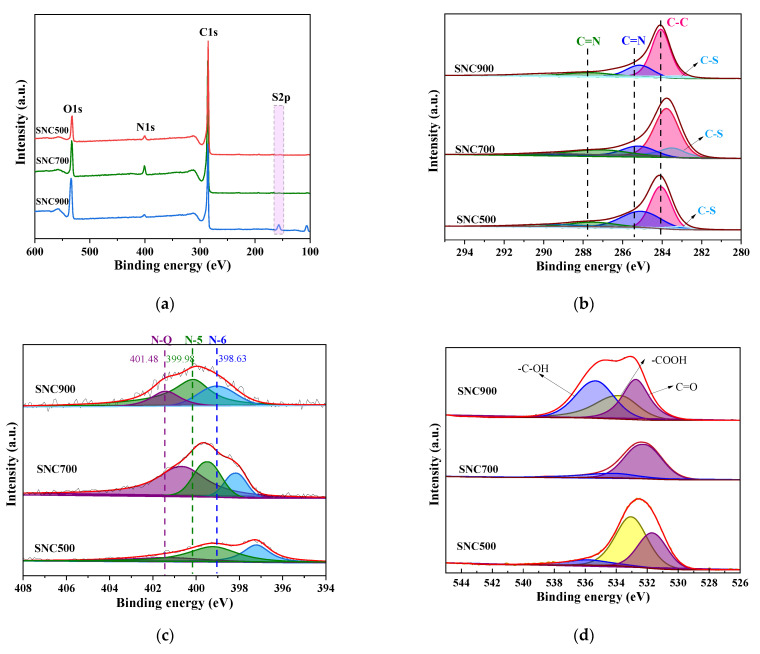

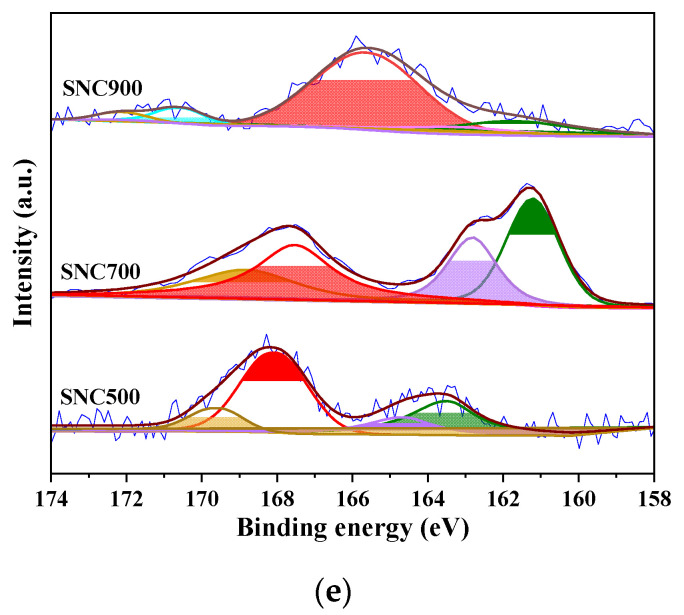
XPS full spectrum (**a**) and C 1s (**b**), N 1s (**c**), O 1s (**d**), and S 2p (**e**) spectra of SNC.

The XPS analysis method is one of the effective means to study the chemical state of elements associated with the surfaces of nanomaterials. To understand the elemental composition of the resulting samples, the surfaces of SNC materials at different carbonization temperatures were analyzed. The XPS full spectra of SNC500, SNC700, and SNC900 are shown in Figure 7a. SNC contains C, N, O, and S elements, with the S content being low and almost unrecognizable in the full spectra. The contents of the three elements in the carbon materials are listed in Table 1.

In order to analyze the components of the prepared carbon material and determine whether N, S, and O were doped into the material, the C 1s fine spectra were separated into peaks by using Avantage software, as shown in Figure 7b. The small peak at 284.09 eV is attributed to sp^2^ C–C, i.e., graphitic carbon, indicating the presence of graphite in all of the prepared materials, which is consistent with the results of TEM and Raman spectroscopy tests. The other peaks at 283.70, 285.06, and 287.51 eV correspond to C–S, C=N, and C=O, respectively. These indicate strong interactions between carbon, nitrogen, and sulfur dopants, further suggesting that N, S, and O elements are also doped into the carbon material lattice [38]. Of these, SNC700 has the most significant peak area corresponding to C–S, the temperature at which most S elements are embedded in LS. A summary of the contents of the different chemical bonding species in the different elements in the various carbon materials is given in Table 1.

The XPS N 1s fine spectra shown in Figure 7c clearly show the effect of carbonization temperature on the bonding mode of the nitrogen group compounds. The fine structure spectra of carbon materials in the mid-N 1s can be classified into three types of nitrogen, all with bonding energies distributed at 398.63 eV, 399.98 eV, and 401.48 eV, corresponding to pyridine nitrogen (N-6), pyrrole nitrogen (N-5), and graphite nitrogen (N-Q), respectively, where the nitrogen groups improve the wettability and conductivity of the electrode material, enhance ion transport, and contribute to further increasing the electroactive specific surface area of the capacitors. Pyrrole/pyridine-type nitrogen (N-5) and pyridine-type nitrogen (N-6) can provide pseudocapacitance to the electrode material via Equations (10) and (11) [39,40].

(10) $$>{C=\mathrm{NH}+2e}^{-}{+2H}^{+}↔>{\mathrm{CH}–\mathrm{NH}}_{2}$$

(11) $$>N^{+}-O^{-}+e^{-}+H^{+}↔>N–\mathrm{OH}$$

As can be seen in Table 1, each sample contains some amount of pyridine nitrogen, pyrrole nitrogen, and graphite nitrogen. As the carbonization temperature increased, the N content of the materials showed an increase followed by a gradual decrease, probably due to the decomposition of C–N, which is unstable at high temperatures. The prepared SNC700 material contains more graphitic nitrogen, while the contents of pyridine nitrogen and pyrrole nitrogen are lower than the contents of the other two materials. Graphitic nitrogen can act as an electron acceptor or attract protons or electrons to increase the electrical conductivity of the carbon electrode material and promote nitrogen or adjacent functional group redox reactions, thus further improving the performance of the supercapacitor and hence the best electrical conductivity of SNC700 [41,42]. After testing, the electrical conductivity of SNC was obtained as 3.0755 S/cm, while the electrical conductivity of NC was 1.8307 S/cm.

In addition, the presence of elemental sulfur in the prepared carbon material can be seen in the form of the XPS S 2p fine spectrum in Figure 7d, where the low elemental sulfur content makes the corresponding characteristic peaks weak. Sulfur is present in the carbon material in two chemical forms: the peak near 168.11 eV corresponds to sulfur oxide (–C–SO_x_–C–) (x = 2, 3, or 4), and the characteristic peaks at 163.52 and 164.53 eV correspond to thiophene sulfur (C–S). The electronegativity of the carbon atom (2.55) is very close to that of sulfur, and the addition of sulfur to the carbon matrix leads to the creation of defects that help to improve the electrochemical properties of the material. The doping of the lignosulfonate effectively binds the sulfur to the carbon lattice, and the sulfur acts as an active site contributing to the redox reaction. However, there is a significant reduction in the relative sulfur content due to the severe heterocyclic ring breakage that occurs during pyrolysis of the molecule [43,44].

In the XPS O 1s spectra shown in Figure 7e, the samples all have three peaks at BE values corresponding to C=O (532.19 eV), –CO (533.03 eV), and –OH (535.49 eV). It can be seen that the oxygen element in all three SNC materials is mainly attached to the carbon atom, and the oxygen-containing groups improve the surface wettability of the carbon material while also reacting reversibly with H^+^ in the acid solution, which in turn improves the electrochemical properties and capacitance value of the material. 

#### 3.1.6. BET Specific Surface Area and Pore Size Analysis

The Brunauer–Emmett–Teller (BET) specific surface areas and pore volume parameters of the porous carbon are summarized in Table 2.

**Figure 8:**
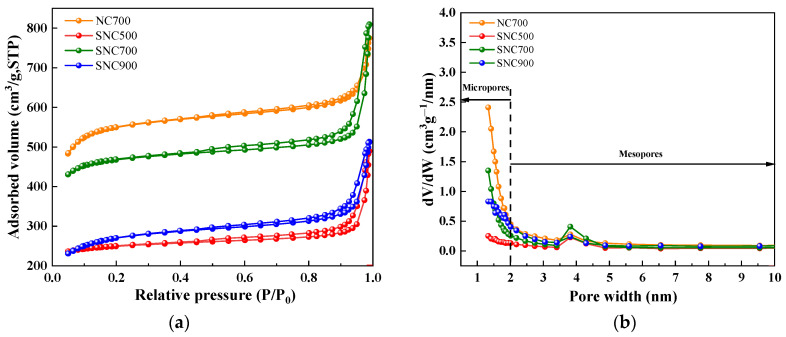
N_2_ adsorption–desorption curves of NC and SNC carbon materials (**a**); mesoporous and microporous pore size distribution of carbon materials (**b**).

The pore size distribution is essential for the application of porous carbon materials. SNC was tested for the adsorption–desorption of N_2_, and the pore size distribution was calculated using the BJH method; the results are shown in Figure 8a,b. As can be seen in the figure, according to the classification system of the International Union of Pure and Applied Chemistry (IUPAC), 4 g of LS was added during the synthesis process. The isotherms of the carbon materials at three carbonization temperatures of 500 °C, 700 °C, and 900 °C were all typical type IV adsorption–desorption isotherms [45], where the higher the relative pressure, the higher the adsorption: i.e., the material showed a pore-filled state. At higher relative pressures, there is a hysteresis loop between the adsorption and desorption branches, indicating the presence of mesopores in the porous carbon [46]. This result is consistent with the pore size distribution curves of the samples in Figure 8b. The SNC700 hysteresis loop has the largest area with the most extensive distribution of mesopores (5–20 nm) and macropores; the mesopores and macropores provide channels for the ions to rapidly shuttle back and forth, resulting in higher rates. Combined with Figure 8b, it can be seen that the most significant proportion of pores in all four materials are micropores (2–5 nm), while in the range of P/P_0_ = 0.1–0.5, the SNC700 adsorption slope is the largest according to the inflection point characteristics of several materials, indicating a gradual widening of the pores [47].

KOH, as an activator, can be effectively and irreversibly inserted into the carbon lattice and swollen carbon lattice for effective etching. After washing, these pores are exposed, resulting in many micropores and small mesopores in the carbon matrix, leading to a significant change in specific surface area and pore size distribution [48].

As can be seen in Table 2, the specific surface areas of SNC are all smaller than those of NC, indicating that as the carbonization temperature is further increased, lignosulfonate does not act as a hard template during the carbonization process but instead sheds unstable non-carbon components (O, H, N, S, etc.) after high-temperature pyrolysis in a N_2_ atmosphere. When the carbonization temperature was increased from 500 °C to 700 °C, the specific surface area of SNC increased from 184.3 m^2^/g to 511.4 m^2^/g. When the carbonization temperature was further increased to 900 °C, it caused the collapse of part of the micro- and mesopore structure. As a result, the specific surface area decreased to 383.2 m^2^/g, and the pore size of micropores decreased from 0.26 cm^3^/g to 0.12 cm^3^/g, with micropores occupying the most significant proportion of the total pores. 

### 3.2. Electrochemical Performance Analysis (Three-Electrode System)

The electrochemical properties of NC and SNC were evaluated by CV, GCD, and EIS. Electrochemical measurements were performed in 6 M KOH solution using a three-electrode system with a potential window of −1.0 to 0 V.

#### 3.2.1. Cyclic Voltammetry

**Figure 9:**
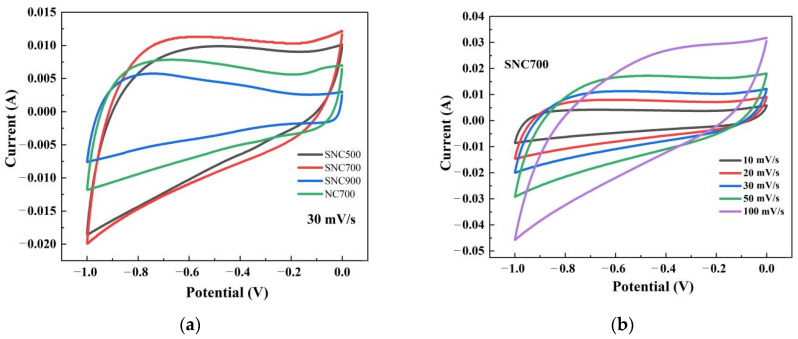
NC and SNC cyclic voltammetry curves at 30 mV/s scanning rate (**a**); cyclic voltammetry curves of SNC700 at different scanning rates (**b**).

The analysis of the morphology and structure of the carbon material under the above different carbonization temperature conditions showed that the prepared SNC700 has a large pore volume and specific surface area and is rich in nitrogen, and the doping of LS further introduces sulfur, which can be used as an electrode material for supercapacitors. The cyclic voltammetry curves of all SNC materials measured according to cyclic voltammetry in the voltage interval of (−1)-0V are shown in Figure 9a. The CV curves for all carbon materials have a more distorted quasi-rectangular shape, which may be caused by the introduction of heteroatoms such as N and S into the carbon material, creating a pseudocapacitance in the 6 M KOH. The size of the cyclic voltammetric area reflects the magnitude of the specific capacitance of the material. The CV area of porous carbon SNC700 is more significant than that of the SNC900 and SNC500 samples, indicating that porous SNC700 has a larger specific capacitance [49]. Figure 9b shows that the area of the CV curve gradually increases as the sweep speed increases, and its shape remains essentially the same, indicating that the SNC material has reasonable charge and discharge characteristics [50].

#### 3.2.2. Constant Current Charge and Discharge

**Figure 10:**
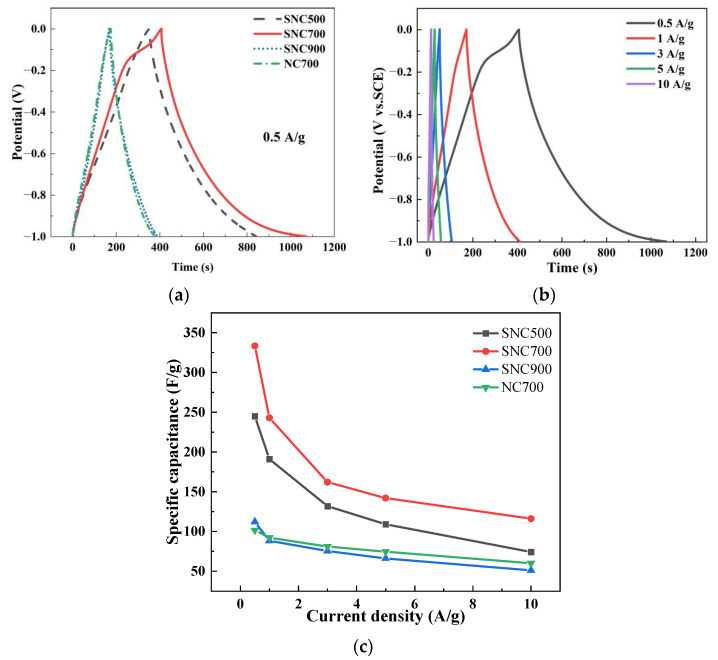
GCD curve of SNC material at 0.5 A/g current density (**a**); galvanostatic charge/discharge curves of SNC700 at different current densities (**b**); specific capacitance of NC and SNC materials at different current densities (**c**).

The electrochemical properties of the NC and SNC materials were investigated using the GCD method, as shown in Figure 10a. At high current charge and discharge densities, the charge and discharge curves of all SNC materials were approximately triangular, showing good double-layer capacitor characteristics [51]. Compared to NC-700, the GCD curve of SNC700 has a concave section at the beginning of the discharge, after which the discharge rate becomes slower than before, indicating that a Faraday reaction occurs during the discharge process, thus further confirming that S doping does provide a Faraday pseudocapacitance for SNC700.

The specific capacitances of all NC and SNC materials at different charge and discharge current densities were calculated according to Equation (2), as shown in Figure 10a. As the charge/discharge current density increases, the specific capacitance gradually decreases (Figure 10c). However, the specific capacitance of the carbon material is still higher than 100 F/g at large charge/discharge current densities, which indicates that the carbon material has good capacitance retention capability (Figure 10b). Among all SNC materials, the SNC700 material has the highest specific capacitance at the same charge/discharge current density, with a high specific capacitance of 333.50 F/g at a charge/discharge current density of 0.5 A/g. NC700 can only reach 101.50 F/g, and even when the charge/discharge current density increases to 10 A/g, its specific capacitance can still reach 116.00 F/g, which is attributed to the high specific surface and relatively abundant N and S contents, and the trend of GCD specific capacitance remains consistent with the CV results.

The above study demonstrates that the specific capacitance of the prepared carbon material mainly originates from the following aspects: (1) with a more significant pore size, mesopores act as fast channels for electrolyte transport/diffusion, which facilitates the smaller pore-size micropores, which can quickly contact the electrolyte, thus enhancing the capacity of the double-layer capacitor; (2) due to the attraction of N and S atoms in the carbon material, the generation of pseudocapacitance can increase the capacity of the capacitor; and (3) the N doped in carbon materials can act as electron acceptors or attract neutrons to improve the charge mobility, which can promote nitrogen or adjacent functional group redox reactions to improve the performance of supercapacitors [6,52].

#### 3.2.3. Cyclic Stability

**Figure 11:**
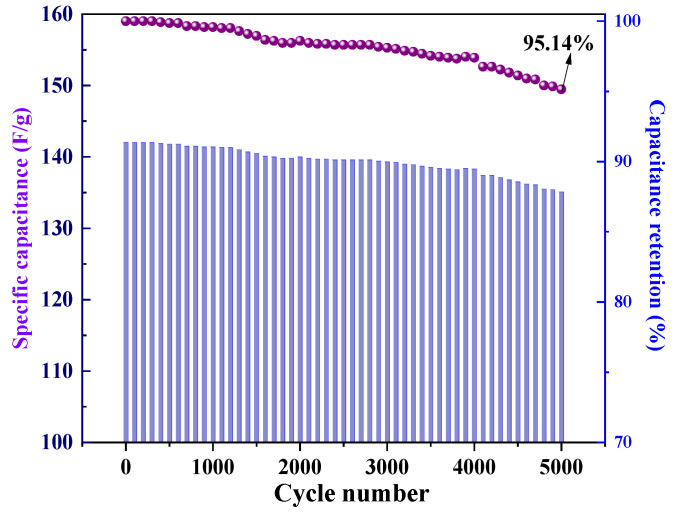
Specific capacitance and capacitance retention of SNC700 electrode material for 5000 cycles.

One characteristic that is essential for the application of porous carbon materials to electrochemical supercapacitors is cycle life. As shown in Figure 11, the SNC700 electrode exhibited good cycling stability at high current density with a potential window of (−1)–0 V. After 5000 charge/discharge cycles at a charge/discharge current density of 5 A/g, the capacity was approximately 95.14% of the initial capacity, reflecting its good long-term stability and demonstrating the effectiveness of heteroatom doping [17]. This provides a potential practical application for carbon materials in supercapacitors.

#### 3.2.4. Electrochemical Impedance Spectra

**Figure 12:**
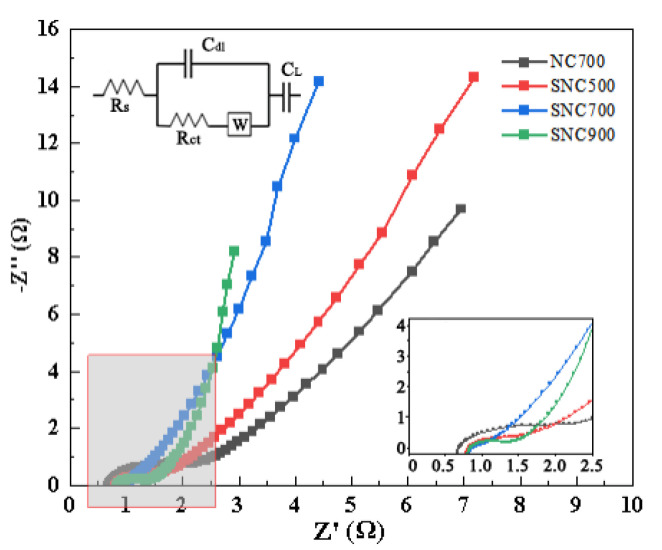
Electrochemical impedance spectra of SNC and NC at different carbonization temperatures (The equivalent circuit diagram of this electrode is shown in the upper left corner of the figure).

An alternating current impedance test (EIS) was performed on the porous carbon material, as shown in Figure 12. Electrochemical impedance spectroscopy has been widely used to study the redox processes of electrode materials, the internal resistance of composites, and charge transfer kinetics and to assess their ionic and electronic conductivity during diffusion at the electrode/electrolyte interface. The Nyquist plots of NC and SNC show a small approximate semicircle in the high-frequency region, followed by a transition to linearity in the low-frequency region, which is typical of carbon materials [53]. Furthermore, the value of the semicircle diameter on the real axis is approximately equal to the charge transfer resistance. In the Nyquist plot, for the four samples mentioned above, SNC700 has a minor intersection point corresponding to the *x*-axis. The charge transfer resistance (*R_ct_*) of the SNC700 electrode is much smaller than that of NC700. The results show that SNC700 has excellent electron transfer and low impedance compared to other electrode materials. This is due to the reversible redox kinetics promoted by the hierarchical porous structure of carbon materials and N and S incorporation. In the mid-frequency region, the 45° line is typical of Warburg diffusion, which is due to the frequency dependence of ion diffusion/transport in the electrolyte. In the low-frequency region, the Nyquist diagram of the SNC electrode shows an imaginary part along with the impedance that is almost perpendicular to the actual axis compared to NC700. These results further confirm that NC and SNC have the shortest ion diffusion distance in the electrolyte [54].

### 3.3. Electrochemical Performance Analysis (Two-Electrode System)

In order to further investigate the feasibility of the SNC700 sample as a supercapacitor electrode material, the NC and SNC samples were assembled into a symmetrical liquid two-electrode system and then subjected to CV and GCD tests [55]. Two NC or SNC carbon material electrode sheets with equal mass were assembled into a button-type symmetric supercapacitor in the order of the battery case, positive electrode, separator, negative electrode, and battery case. Electrochemical measurements were performed in a 6 M KOH solution with a potential window of 0 to 1 V. After that, the two-electrode system was tested.

#### 3.3.1. Cyclic Voltammetry

**Figure 13:**
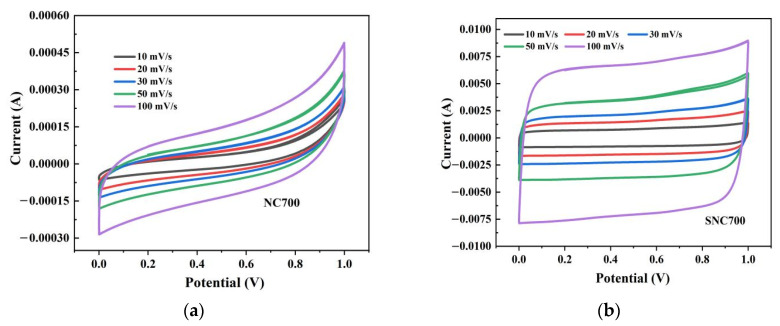
CV curves at different scanning rates ranging from 10 to 100 mV/s for NC700 (**a**) and SNC700 (**b**).

The CV curves of NC700 and SNC700 at scan rates of 10, 20, 30, 50, and 100 mV/s are shown in Figure 13. As the scan rate increases, the CV curve of the device remains basically unchanged, indicating that the device has excellent rate capability. At the same time, the curve of SN700 presents a standard rectangular curve, indicating that the supercapacitor has capacitive performance. As can be observed, the curves of the SNC700 electrode maintain a quasi-rectangular voltammogram shape even at 100 mV/s, indicating its excellent supercapacitive behavior [56]. Furthermore, as can be seen in Figure 13, NC700 has a much lower capacitance than the SNC700 cell, indicating that NC700 is not conductive enough to produce a supercapacitor with sufficient performance. Improvements that validate this work are of great interest.

#### 3.3.2. Galvanostatic Current Charge and Discharge

**Figure 14:**
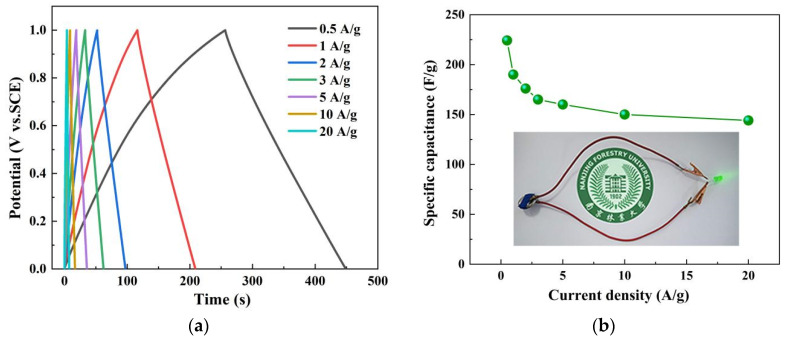
Galvanostatic charge/discharge curves of SNC700 at current densities from 0.5 to 20 A/g (**a**); gravimetric specific capacitances at various current densities (inset photo of the supercapacitor lighting up the LED) (**b**).

As can be seen in Figure 14a, the discharge curve of the SNC700 sample is symmetrical concerning the charging curve and close to a regular isosceles triangle shape, reflecting good electrochemical reversibility and high Coulomb efficiency, indicating its double-layer capacitor characteristics [52,57]. In addition, the GCD curves show negligible IR drops, suggesting low device resistance. The specific capacitance of the SNC700 sample is 224.2 F/g at a current density of 0.5 A/g in the two-electrode system, as calculated by Equation (2). The specific capacitance of the device is shown in Figure 14b. As the current density increases to 0.5, 1, 2, 3, 5, 10, and 20 A/g, the corresponding specific capacitance reaches 224.2, 190.1, 176.2, 165.5, 159.8, 150.1, and 144.0 F/g, respectively. Furthermore, the assembled button supercapacitor can easily light up the LED (below the curve in Figure 14b).

#### 3.3.3. Cyclic Stability and Ragone Plot 

**Figure 15:**
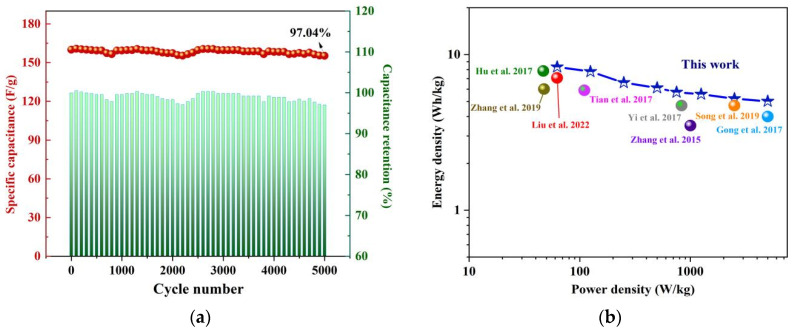
Cyclic stability of symmetric supercapacitors based on SNC700 (**a**); Ragone plot of symmetric supercapacitors based on SNC700 (**b**) [20,45,52,58,59,60,61,62].

The cycling durability of SNC700 was investigated by GCD measurements at a charge/discharge current density of 5.0 A/g for 5000 cycles (shown in Figure 15a). The SNC700-assembled devices exhibited excellent cycling stability with high capacitance retention (97.04%). Figure 15b shows the Ragone plot, from which the assembled device outputs a high energy density of 8.33Wh/kg at a power density of 62.5 W/kg. At the ultra-high-power density of 5000W/kg, the energy density remains at 5.01 Wh/kg, which is better than the previous literature [20,45,52,58,59,60,61,62].

## 4. Conclusions

In summary, lignosulfonate was added to polyaniline for composite pre-carbonization by chemical oxidative polymerization. Then, KOH was used to activate high-temperature carbonization to prepare nitrogen–sulfur co-doped porous carbon microspheres, which were then used to prepare supercapacitor electrode materials. The carbonization temperature affects pore formation in the carbon material. When LS was added at 4.0 g and the carbonization temperature was 700 °C, the porous skeleton structure of the carbon material obtained was well maintained, with a specific surface area of 511.00 m^2^/g and an average pore size of 2.75 nm with a three-dimensional linkage structure and graphitized structure. The successful doping of LS into the crystal structure of the carbon material was confirmed by Raman spectroscopy, XRD, and XPS, and the carbon material provided electron-accepting graphitic N and S elements capable of generating lattice defects, which helped to improve the charge mobility and promote the redox reaction of nitrogen or adjacent functional groups. At 0.5 A/g, the specific capacitance reached 333.50 F/g (three-electrode system) and 242.20 F/g (two-electrode system). After 5000 charge/discharge cycles at 5.0 A/g, the material maintained good cycling stability and was able to achieve a capacitance retention of 97.04% (two-electrode system). Symmetrical devices were capable of delivering an energy density of 8.33 Wh/kg at a current density of 0.25 A/g, a value better than commercial devices.

## Figures and Tables

**Table 1:** Content and composition of C, N, O, and S elements at different carbonization temperatures measured by XPS test.

Samples	N Content (at.%)			S Content (at.%)		O Content (at.%)			Content (at.%)			
	N-5	N-6	N-Q	2p_1/2_	2p_2/3_	O-1	O-2	O-3	C	N	S	O
SNC500	29.20	60.12	10.66	15.11	84.88	34.58	54.73	10.68	90.44	5.97	0.56	3.01
SNC700	27.48	17.23	55.23	20.42	79.57	78.98	19.01	1.96	86.98	7.27	1.01	4.72
SNC900	24.95	36.52	38.51	7.56	92.43	31.66	29.35	38.98	86.50	5.61	0.89	6.98

**Table 2:** Comparison table of specific surface area and pore structure properties of carbon materials at different carbonization temperatures.

Sample	S_BET_ (m^2^/g)	V_mic_ (cm^3^/g)	V_tot_ (cm^3^/g)	D_por_ (nm)	V_mic_/V_total_ (%)
NC700	875.0	0.37	1.20	2.27	31.2
SNC500	184.3	0.05	0.76	3.96	59.3
SNC700	511.4	0.26	1.25	2.75	20.5
SNC900	383.2	0.12	0.80	2.72	15.2

S_BET_: BET surface area, V_tot_: total pore volume; V_mic_: micropore volume; and D_por_: mean pore size.

## Data Availability

Data sharing is not applicable to this article as no datasets were generated or analyzed during the current study.
